# Knowledge and Awareness of Cancer Screening Among the Public in Saudi Arabia: A Cross-Sectional Study

**DOI:** 10.7759/cureus.102042

**Published:** 2026-01-21

**Authors:** Wisam Jamal, Eihab Munshi, Hassan A Alharbi, Saeed A Alaryani, Alwalid M Alharbi, Abdalmjeed S Albreki, Battal N Algethami, Ahmed Y Almadani, Hisham A Rizk

**Affiliations:** 1 Department of Surgery, Faculty of Medicine, University of Jeddah, Jeddah, SAU; 2 Department of Medicine, University of Jeddah, Jeddah, SAU

**Keywords:** awareness, cancer, knowledge, saudi arabia, screening

## Abstract

Background

Cancer screening tests discover cancer at early stages, even before symptoms appear. When abnormal tissues or a malignant mass are found early, treatment and cure rates are improved. In later stages, cancer may have grown and metastasized. This study aims to assess knowledge and awareness of cancer screening in Saudi Arabia.

Methods

A community-based cross-sectional study was conducted among the general population of Saudi Arabia, with a sample of 451 participants. Data were collected through an anonymous online survey addressing sociodemographic information and awareness levels regarding cancer screening. Statistical analysis was conducted using IBM SPSS Statistics for Windows, Version 26 (Released 2018; IBM Corp., Armonk, NY, USA), with categorical variables expressed as frequencies and percentages, and numerical data presented as means and standard deviations. The Chi-square test was used to assess correlations, with a p-value of less than 0.05 considered statistically significant.

Results

This study included a total of 451 participants. The most common age group was 18-29 years (n = 373, 82.7%). Males constituted a slightly higher proportion than females (n = 246, 54.5% vs. n = 205, 45.4%). The majority of participants (n = 369, 81.8%) knew that tests exist for various types of cancer and tumors. Additionally, 426 participants (94.5%) agreed that early detection of cancer helps in its treatment, and 311 (69%) agreed that early detection improves treatment outcomes. Regarding awareness of cancers that can be screened for, breast cancer was the most commonly identified (n = 424, 94%), followed by prostate cancer (n = 271, 60.1%) and colon cancer (n = 270, 59.9%). The prevalence of screening was extremely limited, with only 3.5% (n = 16) having undergone colonoscopy or sigmoidoscopy and 4.9% (n = 22) having undergone other forms of colorectal testing. Analysis of the association between participants’ sociodemographic characteristics and knowledge of cancer screening tests revealed significant associations between gender, educational level, and employment status and general knowledge and awareness of cancer screening (p < 0.05). Females were more knowledgeable than males (p < 0.001). Similarly, respondents with higher educational levels and students demonstrated higher knowledge levels than other respondents (p = 0.003 and p = 0.02, respectively).

Conclusions

Awareness of cancer tests and the benefits of early detection was high, particularly for breast, prostate, and colon cancer, yet actual screening rates remained very low. Knowledge was significantly associated with gender, education, and employment status. However, the study is limited by a significant youth bias, as over 80% of participants were aged 18-29, which may affect the generalizability of the findings to older populations who are at higher risk for cancer.

## Introduction

Cancer ranks second as the most common cause of death worldwide, following ischemic heart disease [1]. The International Agency for Research on Cancer (IARC) reported that the global cancer burden had risen to 18.1 million cases and 9.6 million deaths in 2018 [2]. The Global Cancer Observatory, a constituent of the IARC, projects that the future incidence of cancer and cancer-related deaths will rise to 29.5 million cases worldwide and approximately 16.4 million deaths by 2040 [3]. This trend was demonstrated by the World Health Organization (WHO), which indicated that more than 9,000 deaths were caused by cancer in the Kingdom of Saudi Arabia (KSA) in 2014 [4], increasing to 10,518 deaths in 2018 [5]. According to data from the Global Burden of Disease study, colon cancer is the leading cause of cancer-related mortality, accounting for 1.6% of deaths worldwide and 1.43% of deaths in Saudi Arabia. In contrast, breast cancer accounts for 1.09% of deaths worldwide and 0.96% in Saudi Arabia, while prostate cancer accounts for 0.74% of deaths among the global male population and 0.58% among Saudi men.

Numerous preventive strategies and early diagnostic techniques have been suggested in a bid to curb the prevalence of certain forms of cancer [6-10]. Early detection of abnormal tissue, hyperplasia, or cancer through screening modalities increases the chances of treatment or cure at an earlier stage, before the disease manifests itself [11]. When a patient is diagnosed with cancer, it is usually at a later stage, after sufficient cellular growth has occurred to cause symptoms [12]. This may adversely impact cancer treatment and reduce curability rates [12,13]. Therefore, screening tests are undertaken in individuals who have no cancer symptoms [13]. Furthermore, other researchers have established that screening programs undertaken at an early age are economical when compared with non-screening approaches [14,15].

Screening modalities for cancer ought to be affordable, non-invasive, and capable of reducing mortality rates through early diagnosis [10]. Low-dose computed tomography (LDCT) is a screening method advised for adults who are at high risk of developing lung cancer. Mammograms are screening X-rays carried out in asymptomatic women [5]. Screening mammograms aim to detect breast cancer at a stage when it is so small that it cannot be felt by a woman or physician. Early detection of small breast cancers using screening mammography significantly reduces cancer-related mortality and morbidity [5]. Polyps or cancer in the colon can be detected using several tests. These include stool-based tests (including stool DNA and RNA tests), flexible sigmoidoscopy, colonoscopy, and CT colonography [12,14].

It is of crucial importance that the public be familiar with and aware of the various screening methods and programs, which will aid in reducing mortality rates [12]. The aim of this study is to assess the knowledge of the Saudi community regarding these screening programs, an evaluation that would assist health authorities in enhancing health screening programs and campaigns.

## Materials and methods

A community-based cross-sectional study was conducted among the general population of Saudi Arabia, using a sample of 451 participants. Participants were recruited using a convenience sampling method via an anonymous online survey distributed through social media (WhatsApp and Twitter/X). Participants represented the general public and were not selected based on high-risk status for specific cancers. Recruitment was not restricted to guideline-eligible screening age groups, and detailed high-risk eligibility variables (e.g., hereditary cancer syndromes, strong family history, or defined high-risk smoking exposure such as pack-years) were not used to define inclusion. Accordingly, the findings primarily reflect awareness in a general population sample rather than a high-risk cohort. This approach, while efficient for reaching a broad audience, introduces inherent selection bias, as it favors younger, tech-savvy individuals. Data were collected through an anonymous online survey addressing sociodemographic information and awareness of cancer screening. Statistical analysis was conducted using IBM SPSS Statistics for Windows, Version 26 (Released 2018; IBM Corp., Armonk, NY, USA), with categorical variables expressed as percentages and frequencies, and numerical data presented as means and standard deviations. The Chi-square test was used to assess correlations, with degrees of freedom calculated based on contingency table dimensions. Effect sizes for Chi-square tests were calculated using Cramér’s V to assess the strength of associations. A p-value of < 0.05 was considered statistically significant.

For some screening tests, result data were missing for a small number of participants, which accounts for minor discrepancies between the number of individuals reporting having undergone screening and those reporting test results. Follow-up questions regarding test results were analyzed only for participants who answered “Yes” to having undergone the test, and the total number of respondents (N) is indicated, for clarity.

The sample size was calculated to provide 80% power to detect a small-to-medium effect size (Cohen's d = 0.2), with a significance level (alpha) of 0.05, using a two-tailed test. This calculation indicated a required sample size of approximately 400 participants. We ultimately recruited 451 individuals to account for potential incomplete responses and to enhance the statistical power of our study.

Questionnaire development and validation

The study questionnaire was meticulously developed based on an extensive review of current literature concerning cancer screening awareness and knowledge. It was structured into several key domains: sociodemographic characteristics, general knowledge about cancer, awareness of specific cancer screening modalities (e.g., mammography, colonoscopy, and Papanicolaou (Pap) test), and attitudes toward screening practices. To ensure clarity, comprehensibility, and cultural appropriateness, the questionnaire underwent pilot testing with a cohort of 25 individuals from the target population. Feedback from this pilot phase was instrumental in refining question wording and optimizing the survey flow. The internal consistency reliability of the knowledge-based items within the questionnaire was subsequently assessed using Cronbach's alpha, which yielded a coefficient of 0.78, indicating acceptable reliability.

Ethical considerations

The study protocol received full ethical approval from the Institutional Review Board (IRB) of the University of Jeddah (no. HAP-02-J-094) Research Ethics Committee. Prior to participation, all prospective respondents were directed to a dedicated informed consent page. This page provided a comprehensive overview of the study, explicitly detailing its objectives, the voluntary nature of participation, the estimated time commitment for survey completion, and a firm assurance of the anonymity and confidentiality of all submitted responses. Participants were also provided with contact information for the principal investigator, should they have any questions or concerns. By proceeding to complete the online questionnaire, participants were understood to have provided their implied consent to participate in the study. No personally identifiable information was collected, further safeguarding the privacy and anonymity of all participants. Statistical analysis was conducted using IBM SPSS Statistics for Windows, Version 26 (Released 2018; IBM Corp., Armonk, NY, USA), with categorical variables expressed as percentages and frequencies, and numerical data presented as means and standard deviations. The Chi-square test was used to assess correlations, with a statistically significant p-value of less than 0.05.

## Results

This study included a total of 451 participants. The sociodemographic characteristics of the study population are detailed in Table 1. Briefly, the most prevalent age group was 18-29 years (n = 373, 82.7%), and males constituted a slightly higher percentage than females (n = 246, 54.5% vs. n = 205, 45.4%).

**Table 1:** Sociodemographic characteristics of study participants (n = 451). Abbreviations: DM, diabetes mellitus; PCOS, polycystic ovary syndrome; IBS, irritable bowel syndrome; SD, standard deviation

Sociodemographic	Subgroups	Frequency	Percent
Age in years	Less than 18	18	4.0
	18-29	373	82.7
	30-39	39	8.6
	40-49	11	2.4
	50-59	9	2.0
	60 and more	1	0.2
Gender	Male	246	54.5
	Female	205	45.5
Nationality	Saudi	421	93.3
	Non-Saudi	30	6.7
Social status	Single	405	89.8
	Married	37	8.2
	Divorced	8	1.8
	Widowed	1	0.2
Education level	Not educated	3	0.7
	Primary	2	0.4
	Secondary	108	23.9
	Diploma	54	12.0
	Bachelor	268	59.4
	Postgraduate studies	16	3.5
Employment status	Student	201	44.6
	Full-time employee	136	30.2
	Part-time employee	14	3.1
	Retired	6	1.3
	Free worker/freelancer	22	4.9
	Non-employee	72	16.0
Region	North	37	8.2
	South	64	14.2
	East	85	18.8
	West	107	23.7
	Middle	158	35.0
Do you suffer from any chronic diseases?	Yes	49	10.9
	No	399	89.1
Comorbidities (N = 49)	Asthma	22	44.9
	Hypertension	9	18.4
	DM	7	14.3
	PCOS	4	8.2
	Chronic diseases	4	8.2
	Psychiatric disease	3	6.1
	Thyroid disease	2	4.1
	Anemia	1	2.0
	Heart disease	1	2.0
	IBS	1	2.0
	Others	6	16.3
Height in cm	167.1 ± 9.6	-	-
Weight in Kg	67.2 ± 18.4	-	-

Regarding knowledge and awareness, a significant majority of participants (n = 369, 81.8%) were aware of tests for various types of cancer and tumors. Furthermore, 94.5% (n = 426) agreed that early detection aids in cancer treatment, and 69% (n = 311) believed it improves treatment outcomes. Breast cancer was the most recognized cancer for screening (n = 424, 94%), followed by prostate (n = 271, 60.1%), and colon cancer (n = 270, 59.9%).

Despite high awareness, the prevalence of actual screening uptake was notably low. For colorectal cancer, only 3.5% (n = 16) of all participants (N = 451) reported having undergone colonoscopy or sigmoidoscopy (95% CI: 1.8%-5.3%), and 4.9% (n = 22) had other forms of colorectal testing (95% CI: 2.9%-6.9%). However, these screening prevalence estimates were calculated using the entire study population as the denominator; given that the cohort was predominantly young, they should not be interpreted as screening coverage among age-eligible individuals. Among female participants (n = 205), mammogram uptake was 4.4% (n = 9; 95% CI: 1.6%-7.2%), other breast tests were 7.3% (n = 15; 95% CI: 3.8%-10.9%), and Pap test uptake was 2.4% (n = 5; 95% CI: 0.3%-4.6%). This low uptake, particularly for age-specific screenings like mammograms, is largely attributable to the predominantly young demographic of our study population, in which the majority (82.7%) were aged 18-29 years, and only a small proportion (n = 21, 4.6%) were aged 40 years or older, the recommended age for many screenings.

Accordingly, age-stratified prevalence (e.g., among participants aged ≥40 years for colorectal and breast cancer screening) provides a more appropriate estimate of uptake in the eligible population, and some screening among younger participants may have occurred due to high-risk indications, such as family history, which were not separately assessed in this analysis.

Associations between sociodemographic characteristics and knowledge regarding cancer screening tests were investigated. A significant association was found between gender, educational level, and employment status with general knowledge and awareness (p < 0.05). Specifically, females demonstrated significantly higher knowledge than males (p < 0.001), and respondents with higher educational levels and students also exhibited a higher level of knowledge compared with other groups (p = 0.003 and 0.02, respectively).​​

Overall knowledge and awareness

**Figure 1:**
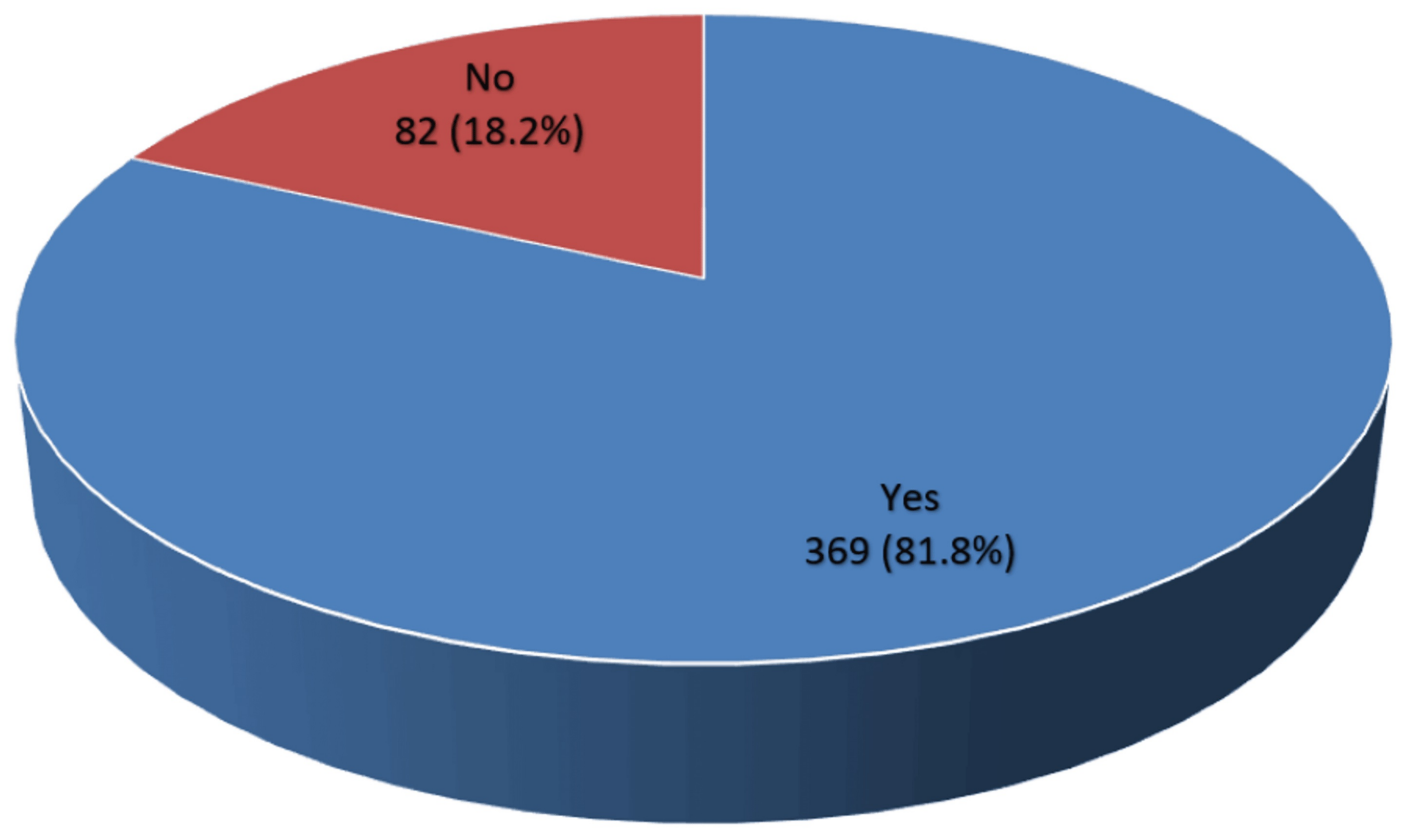
Knowledge regarding tests used for different types of cancer and tumors (n = 451).

The majority of participants (n = 369, 81.8%) knew that there are tests for various types of cancer and tumors (Figure 1).

**Figure 2:**
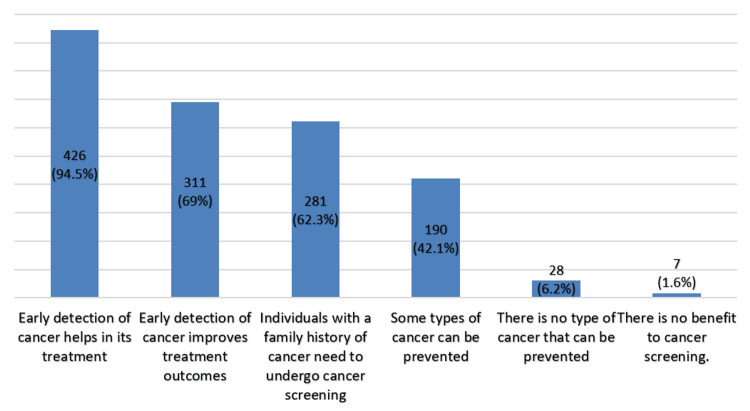
Benefits of cancer and tumor screening (n = 451).

Additionally, 94.5% (n = 426) agreed that early detection of cancer helps in its treatment, and 69% (n = 311) agreed that early detection helps in improving treatment outcomes (Figure 2). 

**Figure 3:**
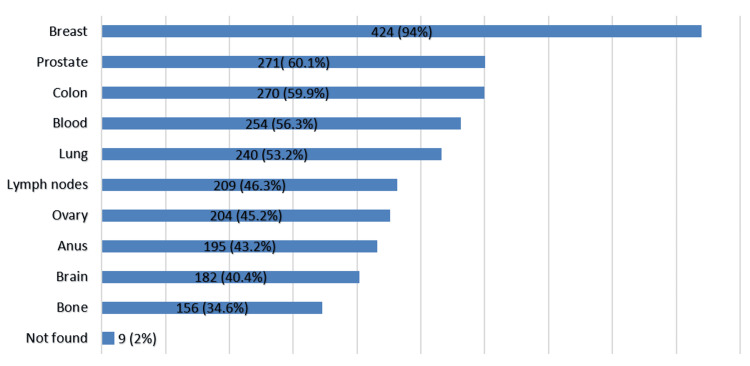
Types of cancer can be screened for (n = 451).

Awareness of cancers that can be screened for reported breast cancer as the most known (n = 424, 94%), followed by prostate (n = 271, 60.1%) and colon cancer (n = 270, 59.9%) (Figure 3).

**Figure 4:**
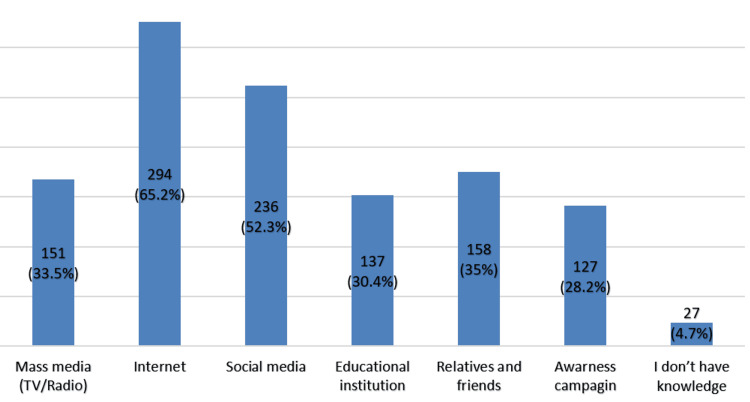
Sources of information regarding tumors and screening (n = 451).

Sources of information varied among participants, with the internet (n = 294, 65.2%) and social media (n = 236, 52.3%) being the most popular (Figure 4).

Knowledge regarding breast cancer screening

Over half of respondents, 53.2% (n = 109), correctly identified age 40 as the recommended starting age for mammography. Additionally, mammography was recognized by 39.5% (n = 81) as the best approved screening method; only 4.4% (n = 9) reported having undergone a mammogram, and 7.3% (n = 15) reported other breast cancer-related tests. This low proportion reflects the calculation across all female participants, the majority of whom were below the recommended screening age for mammography. Among those screened, most results were negative (n = 7, 77.8% for mammography and n = 11, 73.3% for other breast cancer tests). Two-thirds of participants (n = 140, 68.3%) showed willingness to visit the doctor and discuss the possibility of undergoing preventive breast screening (Table 2).

**Table 2:** Knowledge regarding breast cancer screening (n = 205). Abbreviation: MRI, magnetic resonance imaging

Statement	Answer	Frequency	Percent
At what age is it recommended to perform breast cancer screening?	30	62	30.2
	40	109	53.2
	50	9	4.4
	I don’t know	25	12.2
What is the best and approved method for breast cancer screening?	Clinical breast examination	66	32.2
	Breast MRI	16	7.8
	Mammogram	81	39.5
	I don’t know	42	20.5
Have you ever had a mammogram?	Yes	9	4.4
	No	196	95.6
If the answer is "Yes," what was the result of your screening? (N = 9)	Positive	2	22.2
	Negative	7	77.8
Have you had any other breast cancer tests (such as breast MRI, clinical breast examination)?	Yes	15	7.3
	No	190	92.7
If the answer is "Yes," what was the result of your test? (N = 15)	Positive	4	26.7
	Negative	11	73.3
Recommendations indicate that screening should start from age 40 to 74 using a chest X-ray "mammogram" every two years. Do you have the desire and willingness to visit your doctor and discuss the possibility of undergoing preventive breast screening?	Yes	140	68.3
	No	65	31.7

Knowledge regarding cervical cancer screening

Only 30.7% (n = 63) of participants correctly identified age 21 as the recommended starting point for cervical cancer screening. Knowledge of the approved screening methods was also limited, with only 43.4% (n = 89) identifying human papillomavirus (HPV) Pap smear as the best method, while over one-third were unsure (n = 77, 37.6%). When calculated across all female participants, reported cervical cancer screening uptake appeared low; however, many respondents were younger than the recommended screening age, which likely influenced this finding. Only 2.4% (n = 5) had ever undergone a Pap test, and 2.9% (n = 6) had undergone other forms of cervical cancer testing. Among the small number of participants who reported undergoing testing, most reported positive results (n = 4, 80.0%); however, this finding should be interpreted cautiously due to the limited number of screened individuals. Regarding willingness to engage in preventive care, 65.4% (n = 134) expressed readiness to consult their doctor about HPV vaccination, and 69.3% (n = 142) were open to discussing recommended screening based on age and health status (Table 3).

**Table 3:** Knowledge regarding cervical cancer screening (n = 205). Abbreviations: HPV, human papillomavirus; Pap test, Papanicolaou test

Statement	Answer	Frequency	Percent
At what age is it recommended to undergo cervical cancer screening?	21	63	30.7
	31	34	16.6
	41	28	13.7
	51	15	7.3
	I don’t know	65	31.7
What is the best and approved method for screening for cervical cancer?	HPV pap smear	89	43.4
	Pap test smear‎\cervical cytology	25	12.2
	Endocervical sampling	14	6.8
	I don’t know	77	37.6
Have you ever had a cervical smear (Pap test) done?	Yes	5	2.4
	No	200	97.6
Have you undergone any other tests for cervical cancer (for example: cervical cytology, endocervical biopsy/sampling)?	Yes	6	2.9
	No	199	97.1
If the answer is "Yes," what was the result of the test? (N = 5)	Positive	4	80.0
	Negative	1	20.0
The recommendations indicate that cervical cancer can be prevented through the HPV (human papillomavirus) vaccine for children as part of the national immunization schedule, with additional doses between the ages of 9-13 and another series between ages 27-45. Do you have the willingness and readiness to visit your doctor and discuss the recommended vaccine doses and schedule based on your age and health status?	Yes	134	65.4
	No	71	34.6
The recommendations indicate that screening should be done from age 21 to 65 using various methods based on age and health status. Do you have the willingness and readiness to visit your doctor and discuss the possibility of undergoing preventive and diagnostic screenings?	Yes	142	69.3
	No	63	30.7

Knowledge regarding prostate cancer screening

Data showed that 30.9% (n = 76) of participants identified 45 years as the recommended prostate screening age, while 35.8% (n = 88) were not sure. Awareness of approved screening methods was also limited, with only 25.2% (n = 62) selecting the prostate-specific antigen (PSA) test as the best method, while half of the respondents were uncertain. When calculated across all male participants, only 4.1% (n = 10) reported having undergone a PSA test. Among those who reported being screened, 22.2% of participants (n = 2) had positive results, while 77.8% (n = 7) had negative results. However, most participants were below the recommended age for prostate cancer screening. A digital rectal examination was reported by 4.1% (n = 10) of participants, with the majority yielding negative findings (n = 6, 85.7%). Prostate biopsy rates were almost negligible, with only 1.2% (n = 3) having undergone the procedure, of whom 66.7% (n = 2) had positive results. Only 44.7% (n = 110) expressed readiness to consult a doctor about PSA testing, while 55.3% (n = 136) were not ready for this (Table 4).

**Table 4:** Knowledge regarding prostate cancer screening (n = 246). Abbreviations: PSA, prostate-specific antigen; DRE, digital rectal exam; MRI, magnetic resonance imaging

Statement	Answer	Frequency	Percent
At what age is it recommended to undergo prostate screening?	45	76	30.9
	55	64	26.0
	65	13	5.3
	75	5	2.0
	I don’t know	88	35.8
What is the best and approved method for screening for prostate cancer?	DRE	39	15.9
	PSA	62	25.2
	Prostate MRI	21	8.5
	I don’t know	124	50.4
Have you ever had a Prostate-Specific Antigen (PSA) test?	Yes	10	4.1
	No	236	95.9
If the answer is “Yes,” what was the result of the test? (N = 9)	Positive	2	22.2
	Negative	7	77.8
Have you ever had a digital rectal exam (DRE) of the prostate by a specialist doctor?	Yes	10	4.1
	No	236	95.9
If the answer is “Yes,” what was the result of the examination? (N = 7)	Positive	1	14.3
	Negative	6	85.7
Have you ever had a prostate biopsy?	Yes	3	1.2
	No	243	98.8
If the answer is “Yes,” what was the result of the biopsy? (N = 3)	Positive	2	66.7
	Negative	1	33.3
The recommendations indicate that screening should start between the ages 55 and 69 for those without a personal or family history of colorectal or prostate cancer, using the PSA blood test. Do you have the willingness and readiness to visit your doctor and discuss the possibility of undergoing a PSA test?	Yes	110	44.7
	No	136	55.3

Knowledge regarding colorectal cancer screening

A high percentage of participants (n = 179, 39.7%) did not know the recommended age to begin screening for colorectal cancer. Knowledge of approved screening methods was low, with almost half of the respondents (n = 217, 48.1%) being unsure, while colonoscopy every 10 years was the most frequently selected option (n = 133, 29.5%). When calculated across the entire study population, reported colorectal cancer screening uptake appeared low, with 3.5% (n = 16) of participants having undergone colonoscopy or sigmoidoscopy and 4.9% (n = 22) reporting other forms of colorectal testing. Among respondents who answered the follow-up question regarding test results, 11.4% (n = 5) reported positive results and 88.6% (n = 39) reported negative results; the denominator reflects respondents to the results item rather than exclusively those who reported undergoing testing. Among the 16 participants who reported undergoing lower endoscopy, only 13 provided a response to the follow-up question on test results, of whom 38.5% (n = 5) reported positive findings and 61.5% (n = 8) reported negative findings (denominator = 13 respondents to the results item, not the full n = 16). Similarly, among those who reported other colorectal tests (n = 22), only 16 reported test results, of whom 25.0% (n = 4) were positive and 75.0% (n = 12) were negative (denominator = 16).

However, this finding is strongly influenced by the young age distribution of the sample, as most participants were below the recommended age for colorectal cancer screening. Despite the low uptake of screening tests, half of the respondents (n = 228, 50.6%) expressed willingness to consult a physician regarding colonoscopy or other similar screening methods (Table 5).

**Table 5:** Knowledge regarding colorectal cancer screening (n = 451). Abbreviation: CT, computed tomography

Statement	Answer	Frequency	Percent
What is the approved method for screening for colorectal cancer?	Colonoscopy every 10 years	133	29.5
CT or capsule endoscopy	50	11.1	-
Fecal occult blood yearly	51	11.3	-
I don’t know	217	48.1	-
Have you ever undergone a lower endoscopy (colonoscopy or sigmoidoscopy)?	Yes	16	3.5
No	435	96.5	-
If the answer is “Yes,” what was the result? (N = 13)	Positive	5	38.5
Negative	8	61.5	-
Have you undergone any other tests for colorectal cancer (e.g., faecal occult blood test, sigmoidoscopy)?	Yes	22	4.9
No	429	95.1	-
If the answer is “Yes,” what was the result of the test? (N = 16)	Positive	4	25.0
Negative	12	75.0	-
The recommendations indicate that screening should start from age 40 for those without a personal or family history of colorectal cancer, or 10 years earlier than the age at which a relative was diagnosed, if applicable. Do you have the willingness and readiness to visit your doctor and discuss the possibility of undergoing a colonoscopy or other exploratory colorectal screening?	Yes	228	50.6
No	223	49.4	-

Knowledge regarding lung cancer screening

The vast majority (n = 191, 42.4%) were not sure about the recommended age for lung cancer screening. Knowledge of approved screening methods was similarly limited; only 26.4% (n = 119) correctly identified LDCT as the preferred screening modality, whereas more than half of respondents (n = 239, 53%) were unsure. Only 2.9% (n = 13) had undergone LDCT previously. Regarding willingness to seek screening, 51% (n = 230) expressed readiness to consult a physician about LDCT, while 49.0% (n = 221) were unwilling. Interpretation of LDCT uptake is limited, as eligibility for lung cancer screening depends on both age and smoking history, the latter of which was not assessed in this study (Table 6).

**Table 6:** Knowledge regarding lung cancer screening (n = 451). Abbreviation: CT, computed tomography

Statement	Answer	Frequency	Percent
At what age is it recommended for individuals at higher risk to undergo lung cancer screening?	20	57	12.6
	30	64	14.2
	40	51	11.3
	50	88	19.5
	I don’t know	191	42.4
What is the best and approved method for screening for lung cancer?	Chest X-ray	70	15.5
	Low-dose CT scan	119	26.4
	Bronchoscopy	23	5.1
	I don’t know	239	53.0
Have you ever undergone a low-dose chest CT scan for screening purposes?	Yes	13	2.9
	No	438	97.1
If the answer is "Yes," what was the result of the scan? (N = 9)	Positive	3	33.7
	Negative	6	66.3
The recommendations indicate that screening should be done from age 50 to 80 for individuals with a history of smoking 20 pack-years over the last 15 years, using a low-dose chest CT scan. Do you have the willingness and readiness to visit your doctor and discuss the possibility of undergoing this exploratory imaging test?	Yes	230	51.0
	No	221	49.0

Screening uptake among participants aged >40 years

Among the study participants who were more than 40 years old (n = 21), the uptake of breast cancer screening was higher than in the overall study population. One-fifth of women (n = 3, 18.8%) reported having undergone mammography, and one-fourth (n = 4, 25.0%) stated they had other breast cancer screening tests, mostly with negative results. A large number of participants (n = 13, 81.3%) reported being ready to talk to a doctor about undergoing breast screening for prevention.

On the other hand, no prostate cancer screening was conducted among male participants who were age-eligible; none of them had previously undergone a PSA blood test, digital rectal examination, or prostate biopsy. However, more than half of the respondents (n = 3, 60.0%) expressed readiness to talk to their physicians about PSA screening.

For colorectal cancer, a group of the oldest participants had undergone lower endoscopy (n = 3, 14.3%), but none had had any other colorectal screening tests. Notwithstanding, half (n = 11, 52.4%) indicated willingness to consult a doctor regarding colonoscopy.

Regarding lung cancer screening, very few participants reported having undergone LDCT screening in the past (n = 2, 9.5%); however, more than half (n = 12, 57.1%) stated they would be willing to talk to a doctor about LDCT. The uptake of lung cancer screening was restricted by eligibility based on smoking history, which was not evaluated in this study (Table 7). 

**Table 7:** Screening uptake among participants aged >40 years (N = 21). Abbreviations: CT, computed tomography; MRI, magnetic resonance imaging; PSA, prostate-specific antigen

Statement	Answer	Frequency	Percent
Have you ever done Mammogram (breast scan)?	No	13	81.3
	Yes	3	18.8
If the answer is "Yes," what were the results of your test? (N = 3)	Negative	2	66.7
	Positive	1	33.3
Have you had any other breast cancer tests (such as Breast MRI, Clinical breast examination)?	No	12	75.0
	Yes	4	25.0
If the answer is "Yes," what were the results of your test? (N = 4)	Negative	2	50.0
	Positive	2	50.0
The recommendations indicate the need for examination starting from age 40 to the age of 74, via chest mammogram every two years. Are you willing and ready to visit your doctor and discuss with him the possibility of having a preventive breast exam?	No	3	18.8
	Yes	13	81.3
Have you had a prostate-specific antigen (PSA) test?	No	5	100.0
	Yes	0	0.0
Have you ever had a rectal prostate exam performed by a specialist?	No	5	100.0
	Yes	0	0.0
Have you had a prostate biopsy?	No	5	100.0
	Yes	0	0.0
The recommendations indicate the need for screening starting from age 55-69 for those who do not have a personal or family history of colorectal cancers, by means of a prostate-specific antigen (PSA) blood test. Are you willing and ready to visit your doctor and discuss with him the possibility of having a prostate-specific antigen (PSA) test?	No	2	40.0
	Yes	3	60.0
Have you ever had a lower endoscopy?	No	18	85.7
	Yes	3	14.3
If the answer is "Yes," what was the result? (N = 3)	Negative	2	66.7
	Positive	1	33.3
Have you had any other tests for colorectal cancer (such as fecal occult blood test, sigmoidoscopy)?	No	21	100.0
	Yes	0	0.0
The recommendations indicate the need for screening starting from the age of 40 for those who do not have a personal or family history of colorectal cancer, and 10 years before the age of diagnosis of a relative if this has occurred. Are you willing and ready to visit your doctor and discuss with him the possibility of having an exploratory colonoscopy?	No	10	47.6
	Yes	11	52.4
Have you ever had a low-dose exploratory chest CT scan?	No	19	90.5
	Yes	2	9.5
If the answer is "Yes," what was the result of the examination? (N = 2)	Negative	2	100.0
	Positive	0	0.0
The recommendations indicate the necessity of screening from age 50 to age 80 for those with a history of smoking 20 packs per year during the last 15 years, using low-dose chest CT scans. Are you willing and ready to visit your doctor and discuss the possibility of undergoing screening scans?	No	9	42.9
	Yes	12	57.1

Screening uptake among participants aged >30 years

In the case of female participants above the age of 30 (n = 60), the prevalence of cervical cancer screening was higher than that of the overall female study population. One out of six respondents (n = 4, 17.4%) reported having a cervical smear, and most of the results were positive (n = 3, 75.0%). A smaller number of women (n = 2, 8.7%) stated that they had undergone related tests, which yielded mixed results. The majority of participants (n = 13, 56.5%) expressed interest in discussing the HPV vaccination with their doctors, whereas more than two-thirds (n = 16, 69.6%) were open to talking about the recommended preventive and diagnostic cervical screening examinations according to their age and health condition (Table 8). 

**Table 8:** Screening uptake among participants aged >30 years (N = 60).

Statement	Answer	Frequency	Percent
Have you ever do necervical smear?	No	19	82.6
	Yes	4	17.4
If the answer is "Yes," what was the result of the swab test? (N = 4)	Negative	1	25.0
	Positive	3	75.0
Have you had any other tests for cervical cancer? (e.g., Papanicolaou test (Pap smear/cervical cytology), endocervical sampling)?	No	21	91.3
	Yes	2	8.7
If the answer is "Yes," what was the result of the analysis? (N = 2)	Negative	1	50.0
	Positive	1	50.0
The recommendations indicate that, God willing, cervical cancer can be prevented by taking a vaccine/medicine - human papillomavirus vaccine. For children within the national immunization schedule, and several other doses between the ages of 9-13, and others between the ages of 27-45. Do you have the desire and willingness to visit your doctor and discuss with him the mechanisms and doses of the recommended vaccine based on your age and health condition?	No	10	43.5
	Yes	13	56.5
The recommendations indicate the need for examination starting from age 21 up to age 65, through several methods based on age and health condition. Are you willing and ready to visit your doctor and discuss with him the possibility of undergoing preventive and exploratory examinations?	No	7	30.4
	Yes	16	69.6

Sociodemographic factors associated with overall knowledge and awareness regarding cancer screening

Investigating the association between sociodemographic characteristics of participants and knowledge regarding tests used for cancer screening revealed a significant association between gender, educational level, and employment status with general knowledge and awareness regarding tests used for cancer (p < 0.05), with females being more knowledgeable than males (p < 0.001). Similarly, respondents with higher educational levels and students had a higher level of knowledge than other respondents (p = 0.003 and 0.02, respectively) (Table 9).

**Table 9:** Sociodemographic factors associated with overall knowledge and awareness regarding cancer screening. *Significant association (effect size calculated using Cramér’s V).

Factors	Subgroups	Do you know that there are tests for various types of cancer and tumors				
		Yes	No	DOF	Effect size	p-value
Age	<18 years	83.3%	16.7%	5	0.05	0.9
	18-29 years	82.0%	18.0%			
	30-39 years	76.9%	23.1%			
	40-49 years	81.8%	18.2%			
	50-59 years	88.9%	11.1%			
	60 and more	100.0%	0			
Gender	Male	74.8%	25.2%	1	0.2	<0.001^*^
	Female	90.2%	9.8%			
Nationality	Saudi	81.9%	18.1%	1	0.01	0.8
	Non-Saudi	80.0%	20.0%			
Social status	Single	81.7%	18.3%	3	0.04	0.8
	Married	83.8%	16.2%			
	Divorced	75.0%	25.0%			
	Widowed	100.0%	0			
Education level	Not educated	66.7%	33.3%	5	0.2	0.003^*^
	Primary	100.0%	0			
	Secondary	79.6%	20.4%			
	Diploma	63.0%	37.0%			
	Bachelor	86.2%	13.8%			
	Postgraduate studies	87.5%	12.5%			
Employment status	Student	87.1%	12.9%	5	0.2	0.02^*^
	Full-time employee	79.4%	20.6%			
	Part-time employee	71.4%	28.6%			
	Retired	83.3%	16.7%			
	Free worker/lancer	59.1%	40.9%			
	Non-employee	80.6%	19.4%			
Region	North	91.9%	8.1%	4	0.1	0.2
	South	85.9%	14.1%			
	East	75.3%	24.7%			
	West	83.2%	16.8%			
	Middle	80.4%	19.6%			
Do you suffer from any chronic diseases?	Yes	85.7%	14.3%	1	0.04	0.5
	No	81.5%	18.5%			

Association between knowledge of tests used for cancer screening and awareness of screening age and utilization of screening tests

The association between awareness of the availability of cancer screening tests and having undergone a mammogram was significant (p = 0.01). Interestingly, participants who had previously undergone mammography demonstrated lower awareness compared to those who had never been screened. Another significant association was found between awareness and knowledge of the recommended age for lung cancer screening among high-risk individuals (p < 0.001). No other significant associations were observed (Table 10).

**Table 10:** Association between knowledge of tests used for cancer screening and awareness with screening age and utilization of screening tests. *Significant association (effect size calculated using Cramér’s V). Abbreviations: PSA, prostate-specific antigen; Pap test, Papanicolaou test; CT, computed tomography

Factors	Subgroups	Do you know that there are tests for various types of cancer and tumors				
		Yes	No	DOF	Effect size	p-value
At what age is it recommended to perform breast cancer screening?	30	85.5%	14.5%	3	0.1	0.3
	40	84.4%	15.6%			
	50	66.7%	33.3%			
	I don’t know	92.0%	8.0%			
Have you ever had a mammogram?	Yes	55.6%	44.4%	1	0.2	0.01^*^
	No	86.2%	13.8%			
At what age is it recommended to undergo cervical cancer screening?	21	93.7%	6.3%	4	0.2	0.2
	31	97.1%	2.9%			
	41	85.7%	14.3%			
	51	93.3%	6.7%			
Have you ever had a cervical smear (Pap test) done?	Yes	100.0%	0	1	0.05	0.5
	No	90.0%	10.0%			
At what age is it recommended to undergo prostate screening?	45	73.7%	26.3%	4	0.2	0.05
	55	81.3%	18.8%			
	65	100.0%	0			
	75	60.0%	40.0%			
	I don’t know	68.2%	31.8%			
Have you ever had a Prostate-Specific Antigen (PSA) test?	Yes	60.0%	40.0%	1	0.07	0.3
	No	75.4%	24.6%			
At what age is it recommended to undergo screening for colorectal cancer?	30	80.9%	19.1%	4	0.1	0.6
	40	89.0%	11.0%			
	50	86.4%	13.6%			
	60	88.2%	11.8%			
	I don’t know	76.0%	24.0%			
Have you ever undergone a lower endoscopy (colonoscopy or sigmoidoscopy)?	Yes	81.3%	18.8%	1	0.003	0.9
	No	81.8%	18.2%			
At what age is it recommended for individuals at higher risk to undergo lung cancer screening?	20	77.2%	22.8%	4	0.2	<0.001^*^
	30	87.5%	12.5%			
	40	78.4%	21.6%			
	50	96.6%	3.4%			
	I don’t know	75.4%	24.6%			
Have you ever undergone a low-dose chest CT scan for screening purposes?	Yes	61.5%	38.5%	1	0.09	0.05
	No	82.4%	17.6%			

## Discussion

This study investigated the public's awareness and knowledge of cancer screening in Saudi Arabia, revealing important insights into current understanding and screening practices. In this study of 451 predominantly young Saudi adults (n = 373, 82.7% aged 18-29), we found a notable disparity between high awareness of cancer screening benefits and significantly low actual screening rates. We observed a high level of awareness about cancer screening in general (81.8% knew screening exists; 94.5% agreed that early detection helps treatment), but very low self-reported uptake of specific cancer screening tests. While this might initially appear as a discordance between knowledge and behavior, it is consistent with patterns seen in previous research, where awareness does not reliably translate into preventive action without addressing structural, psychosocial, and eligibility barriers [16]. It is crucial to consider the demographic characteristics of our cohort.

Given that 82.7% of participants were aged 18-29 years, the low uptake of cancer screening tests should be interpreted cautiously. Many routine screening programs (e.g., mammography, colorectal cancer screening, and prostate cancer screening discussions) primarily target older age groups; therefore, a substantial proportion of our respondents were likely not eligible for routine screening at the time of participation. Accordingly, the low prevalence of self-reported screening in our cohort may reflect guideline ineligibility as much as behavioral or access-related barriers.

A key factor contributing to the low screening rates in our sample is indeed the young age distribution. Many standard screening recommendations, for example, mammography, colorectal screening, and prostate cancer discussions, are primarily targeted at older age groups. Consequently, a substantial portion of the participants in this study would not yet be eligible for routine screening based on age-related guidelines. Therefore, while general awareness is high, the observed low prevalence of test uptake in our cohort is, to a significant extent, an expected outcome given the age profile of our participants, rather than solely a failure of knowledge translation.

Breast cancer was the most widely recognized screening-eligible cancer (n = 424, 94%), followed by prostate (n = 271, 60.1%) and colon cancer (n = 270, 59.9%). This aligns with previous Saudi and regional studies; for example, a large community-based Saudi survey also found that breast cancer is the most recognized cancer for screening [17]. The dominance of breast cancer in public consciousness is likely driven by sustained national public-health messaging and female-centered screening campaigns, which may not yet be mirrored in public awareness of other cancers.

Only 4.4% (n = 9) of participants reported having had a mammogram, and just 2.4% (n = 5) had a Pap smear, while only 4.1% (n = 10) had ever undergone a PSA test. These low rates are concerning, but they reflect broader trends in Saudi Arabia. For instance, in prostate cancer screening, prior studies in the Asir region found good general knowledge (82.5%) but much lower awareness of specific screening methods (49.4%) and actual PSA testing [18]. Similarly, in Najran, only 30.5% were aware of PSA testing, and just 3.2% had seen a specialist [19].

Regarding barriers to screening, the findings suggest that a lack of detailed, actionable knowledge may be one barrier. For cervical cancer screening, only 30.7% (n = 63) correctly identified 21 years as the starting age, and only 43.4% (n = 89) selected HPV Pap smear as the best; over a third were unsure. These gaps mirror international evidence, with low socioeconomic status, lack of physician recommendation, and embarrassment or fear identified as key deterrents [20]. In Saudi Arabia, especially, cultural sensitivity, lack of public campaigns, and limited screening infrastructure have been flagged as obstacles, particularly for colorectal cancer [21,22].

Correlation testing found that females, those with higher education, and students had significantly higher levels of screening knowledge. This aligns with the theory that health literacy, socioeconomic status, and regular exposure to health messaging influence preventive behavior. Cognitive factors (e.g., perceived behavioral control and subjective norms) have also been shown to mediate screening intentions and uptake [21]. These demographic patterns suggest that targeted educational interventions could be beneficial, focusing on groups with lower awareness levels.

While general awareness of screening benefits was high, there were gaps in actionable knowledge (which test to take, when to start, recommended intervals, and where to access services). Public health messaging should, therefore, be clearer, age-specific, and risk-focused, emphasizing evidence-based eligibility criteria and pathways for accessing screening. Targeted educational interventions may be particularly beneficial for groups with lower awareness levels and should be aligned with guideline-eligible age groups and individuals at increased risk.

In conclusion, despite high awareness of cancer screening benefits among predominantly young Saudi adults, uptake of specific screening tests was low, likely reflecting both guideline ineligibility and other barriers. Future efforts should prioritize improving practical knowledge and facilitating access among guideline-eligible populations and high-risk individuals to maximize the effectiveness of screening programs.

Limitations of the study

A major limitation relates to the age distribution of the sample. The study population was predominantly young, with 82.7% of participants aged 18-29 years, while individuals in higher-risk age groups for most cancer screening programs (≥45 years) constituted only a very small proportion of the sample. As a result, the findings regarding screening uptake and eligibility may be skewed toward lower observed screening rates and may not accurately reflect behaviors or knowledge among older adults, who are more likely to meet screening criteria.

In addition, self-reported screening behavior may suffer from recall bias or social desirability bias. Furthermore, the cross-sectional design prevents assessment of causality (e.g., whether knowledge leads to future screening). Finally, the snowball sampling technique may have introduced selection bias. Data collection through an anonymous online survey may also introduce selection bias, as individuals with greater internet access or a predisposition to participate in health-related surveys might be overrepresented.

Future studies should employ age-stratified sampling and longitudinal designs to better assess determinants of screening uptake. Additionally, a limitation of this study is the potential for sampling bias due to the online distribution method via social media platforms and snowball sampling. This approach may have led to an overrepresentation of individuals who are active on these platforms, particularly those in the 18-29 age group and within specific social networks. This demographic characteristic is a notable weakness, as this population typically does not qualify for routine cancer screening, potentially limiting the generalizability of our findings regarding screening awareness and uptake to the broader Saudi population. Future studies could employ stratified sampling or other methods to ensure broader age and demographic representation.

## Conclusions

This study demonstrates that, among a predominantly young adult sample in Saudi Arabia, general awareness of cancer screening and its perceived benefits is relatively high, while detailed knowledge of recommended screening methods, eligibility criteria, and actual screening uptake remains limited. The low prevalence of screening observed should be interpreted in the context of the participants’ young age distribution, as many were not eligible for routine screening according to current guidelines. Sociodemographic factors - particularly gender, education level, and employment status - were significantly associated with awareness levels within this population. Accordingly, these findings are most applicable to younger adults and should not be generalized to guideline-eligible older populations or high-risk groups.

Future research using age-stratified, nationally representative sampling and explicit risk stratification is warranted to better characterize screening knowledge and behaviors across eligible and high-risk populations in Saudi Arabia, and to inform targeted public health interventions.
